# Selected Functional and Processing Properties of Polyhydroxyalkanoates

**DOI:** 10.3390/ma19173587

**Published:** 2026-08-24

**Authors:** Mariusz Fabijański, Jacek Garbarski

**Affiliations:** Plastics Processing Department, Faculty of Mechanical and Industrial Engineering, Warsaw University of Technology, 85 Narbutta Street, 02-524 Warsaw, Poland; jacek.garbarski@pw.edu.pl

**Keywords:** polyhydroxyalkanoates, PHA, biodegradable polymers, mechanical properties, processing

## Abstract

Polyhydroxyalkanoates (PHAs) are biodegradable biopolymers with high application potential, representing an alternative to petrochemical-derived plastics. The aim of this study was to evaluate the processing and mechanical properties of the commercial PHA EM20010 material intended for injection molding. Particular attention was paid to analyzing the effect of process parameters on the material’s flowability in the injection mold and determining the basic functional properties of the resulting molded parts. The tests included a technological flowability test using an Archimedes spiral mold, a static tensile test, Charpy impact testing, Shore D hardness measurements, and water absorption assessment. The results obtained showed that the tested material was characterized by a high tensile strength of 66.8 ± 1.8 MPa and a Young’s modulus of 1322 ± 21 MPa. Elongation at maximum stress reached 5.0 ± 0.5%, indicating the relatively stiff nature of the material. The average unnotched impact strength was 24.8 ± 3.5 kJ/m^2^, while hardness ranged from 54.6 to 55.4 ShD. Water absorption tests revealed very low water absorption of approximately 0.11% after 24 h of exposure. The flushing test confirmed the significant effect of injection temperature and pressure on the material’s flowability. The maximum spiral length of 54.8 cm was achieved at a processing temperature of 175 °C and an injection pressure of 100 bar. The tests demonstrated that this material possesses a favorable combination of high strength, good toughness, low water absorption, and good processing properties.

## 1. Introduction

The contemporary development of the circular economy and the growing pressure to reduce the negative impact of plastic waste on the natural environment are driving the intensification of research on biodegradable materials [1,2]. Bio-based polymers are of particular importance as they can be an alternative to conventional plastics obtained from petrochemical raw materials [3,4,5]. Among the most used and studied biodegradable materials are polylactide (PLA) and thermoplastic starch (TPS). However, polyhydroxyalkanoates (PHAs), distinguished by their unique combination of biodegradability, biocompatibility, and wide possibilities of shaping functional properties [6,7,8,9,10], are attracting increasing scientific and industrial interest.

Polyhydroxyalkanoates are a group of aliphatic polyesters synthesized by numerous species of microorganisms during fermentation processes [11,12,13,14,15]. Under conditions of excess carbon source and limited availability of other nutrients, bacteria accumulate PHAs in the form of intracellular granules, which act as energy and carbon stores [16].

**Figure 1:**
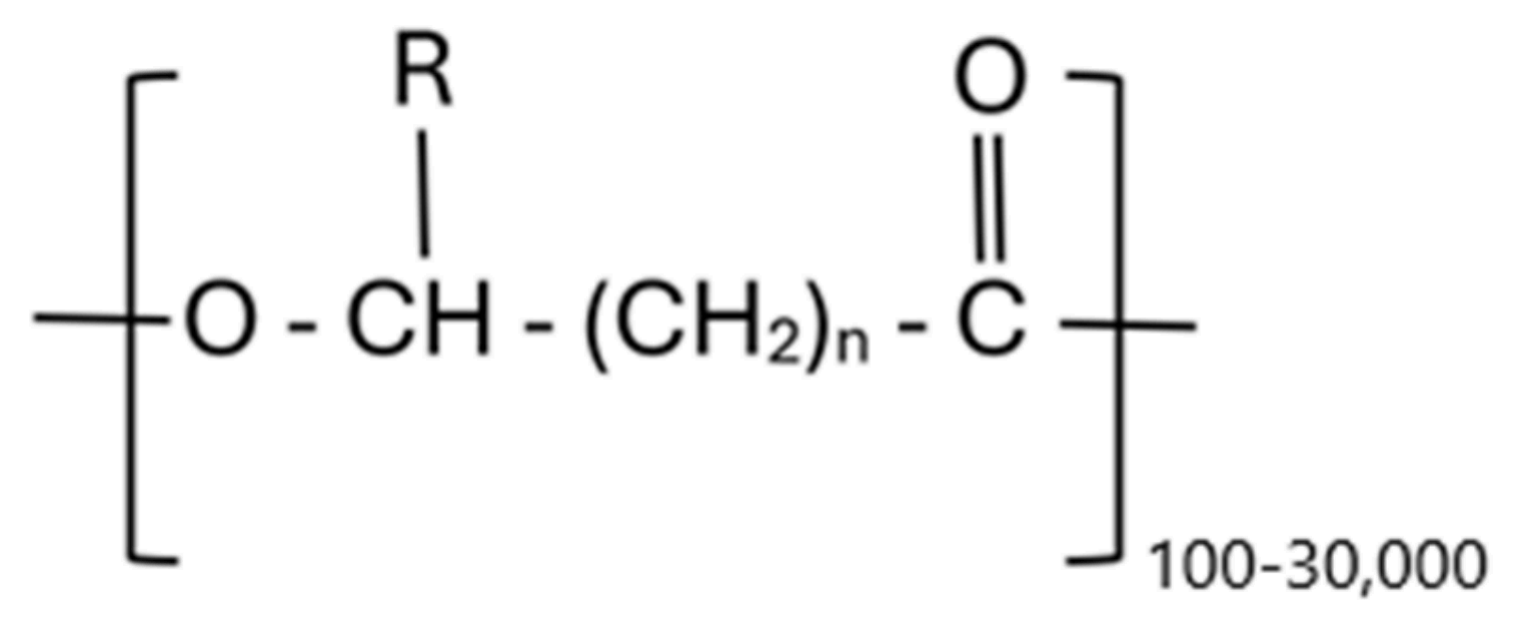
Schematic diagram of the general structure of monomers building PHA chains [26].

**Figure 2:**
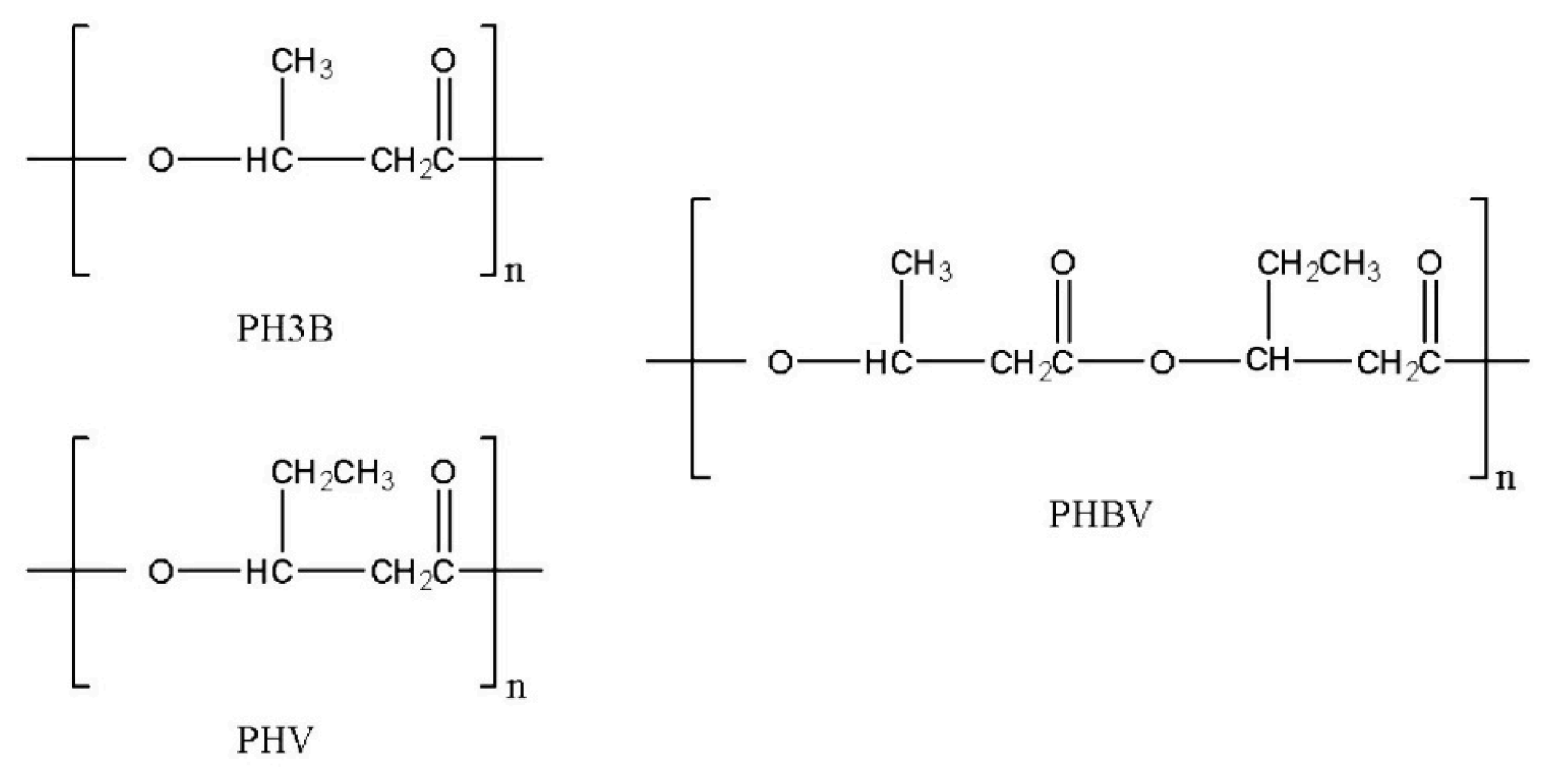
Structural structure of selected polyhydroxyalkanoates [27].

A special feature of this group of materials is the chemical diversity of the monomers that build the polymer chain, which allows the production of materials with diverse mechanical, thermal, rheological, and degradation properties [17,18,19,20,21]. The most important representatives of this group include poly(3-hydroxybutyrate) (PHB), poly(3-hydroxybutyrate-co-3-hydroxyvalerate) (PHBV), and poly(3-hydroxyvalerate) (PHV) [22,23,24]. Figure 1 shows the general scheme of PHA, while Figure 2 shows the general structure of selected polyhydroxyalkanoates [25].

The combination of biodegradability and biocompatibility makes PHAs useful not only in the packaging sector but also in medicine, pharmacy, tissue engineering, agriculture, and the cosmetics industry [28,29,30]. These materials are used, among others, to produce biodegradable implants, tissue scaffolds, controlled drug release systems, surgical sutures, stents, and components intended for tissue regeneration. At the same time, the possibility of complete biodegradation in various environments makes PHAs one of the most promising groups of biopolymers of the future [31,32,33,34,35,36,37,38,39].

Despite numerous advantages, wider implementation of polyhydroxyalkanoates still faces significant technological and economic limitations. High production costs, related to both the biosynthesis process and the extraction and purification of the polymer, pose an additional challenge. The processing properties of many PHA varieties [40,41,42,43]. Crystallinity, narrow processing window, and the possibility of thermal degradation during processing mean that processes such as extrusion, injection molding, thermoforming, and 3D printing require careful optimization of technological parameters. At the same time, processing conditions significantly affect product properties, including mechanical properties [44,45,46,47,48].

The aim of this study was to conduct comprehensive research on the processing of polyhydroxyalkanoates (PHA) and assess the mechanical properties of the resulting injection-molded products. Particular attention was paid to analyzing the relationship between processing parameters and the functional properties of the finished molded parts. A significant novelty of this work was the detailed assessment of the effect of processing parameters on the flowability of molten PHA in the mold cavity. The studies were conducted using an injection mold equipped with an open measuring channel in the shape of an Archimedean spiral, enabling quantitative assessment of the material’s flowability under conditions corresponding to the actual injection molding process.

The obtained results may constitute the basis for the development of optimal conditions for the processing of polyhydroxyalkanoates and contribute to the wider use of these materials in industry, especially in areas requiring a combination of high functional properties with biodegradability and origin from renewable sources.

## 2. Materials and Methods

A commercial biopolymer with the trade name EM20010, manufactured by Ecomann Bioresin (Shenzhen, China), was used in the study. According to the manufacturer, it is a partially crystalline, biodegradable aliphatic biopolyester intended for injection molding [49]. The main component of the material is polyhydroxyalkanoates (PHA). It is doped with polylactide (PLA) as a modifying agent, affecting the processing and functional properties of the composition. The exact composition of the commercial EM20010 grade is proprietary and has not been disclosed by the manufacturer. According to the technical documentation available.

EM20010 belongs to the group of biodegradable thermoplastics that meet the requirements of standards for compostability and biodegradability, including EN 13432. Due to its properties, this material is primarily used in the production of injection molded products, such as personal hygiene products, cosmetic packaging, disposable tableware, trays, and other products intended for short-term use. Table 1 presents the basic specifications of this material.

For injection molding applications susceptible to thermal degradation, precise temperature control is recommended. For example, temperatures in the feed zone are recommended between 140 and 150 °C, in the compression zone between 155 and 165 °C, and in the dispensing zone and at the nozzle, temperatures between 165 and 180 °C are recommended to avoid PHA degradation. Thermostating the injection mold and maintaining its operating temperature between 30 °C and 50 °C is also recommended.

Compared to many other biodegradable materials, this material is characterized by a favorable set of mechanical and functional properties. Its key advantages include high stiffness, high surface gloss, good thermal resistance, very good hydrolysis resistance, and favorable processing properties resulting, among other things, from the material’s high molecular weight [50,51,52].

It should be emphasized, however, that it is not compatible with commonly used conventional polymers, such as polyethylene (PE), polypropylene (PP), poly(ethylene terephthalate) (PET), or polystyrene (PS) [53,54]. At the same time, it can be effectively combined with other biodegradable polymers, in particular polylactide (PLA), poly(butylene succinate) (PBS), poly(butylene adipate-co-succinate) (PBSA), and other biodegradable materials. This creates broad opportunities for designing new material compositions with optimized processing, mechanical, and functional properties [55,56,57].

The test samples were produced by injection molding on a Ponar Żywiec UT90 horizontal screw injection molding machine (Żywiec, Poland), part of the UT series designed for thermoplastics processing. The machine is equipped with a five-point, two-lever mold closing mechanism, ensuring high system stiffness and uniform clamping force distribution. The plasticizing system is based on a 30 mm diameter screw with an L/D ratio of 21, a compression ratio of 2.5, and a maximum injection volume of 106 cm^3^. It is directly driven by a high-torque hydraulic motor, enabling efficient plasticization and stable processing of materials with increased melt viscosity.

The injection process was carried out using a set of peripheral devices adapted to the nature of the research. The material’s processing properties were assessed using a specialized test mold equipped with an Archimedean spiral-shaped measuring socket with an open flow channel. This solution enabled analysis of the impact of injection process parameters on the flowability of the molten polymer and evaluation of its processability under conditions like those in real industrial environments.

To prepare samples for mechanical testing, an injection mold equipped with replaceable mold inserts was used, enabling the production of samples in the form of dumbbells for strength testing and bars for stiffness and impact testing. A thermostat responsible for maintaining the preset injection mold temperature ensured stabilization of the process temperature. During the tests, a RADwag, (Radom Poland) electronic scale was also used to control the mass of the molded parts, and a KC100/200 (Elkon, Dawidy Bankowe, Poland) laboratory dryer was used to prepare the material before processing. Additionally, the test stand was equipped with a plastics recycling mill, enabling the grinding of sprues and defective molded parts for reuse in the processing process.

To comprehensively characterize the polyhydroxyalkanoate under study, mechanical properties were tested. Strength tests were performed using the static tensile method in accordance with ISO 527 [58]. A Heckert Fu1000e machine (Leipzig, Germany) equipped with a 10 kN measuring head was used. Standardized samples were stretched at a constant speed of 2 mm/min, with force and elongation values continuously recorded until fracture.

The material’s resistance to dynamic loads (impact strength) was determined in accordance with ISO 179-1 [59] using the Charpy method. Measurements were performed using a pendulum impactor from Wolfgang Ohst (Rathenow, Germany). The impact energy recorded during the test was used to calculate the final impact strength of the tested samples.

The hardness test was carried out using the Shore method (D scale) based on the guidelines of the ISO 868 [60] standard. An electronic hardness tester from XINGWEIQIANG (Huizhou, Guangdong, China) was used for the measurements.

Water absorption tests were also carried out during 24 h of storage of samples in an aqueous environment, based on the guidelines of the ISO 62 standard. To precisely determine the mass increase, an analytical balance from Radwag (Radom, Poland) was used.

The functional properties tests were supplemented by an analysis of the material’s processing properties. For this purpose, a technological flowability test was conducted to assess the impact of basic injection process parameters, particularly processing temperature and injection pressure, on the flowability of the molten polymer in the mold cavity. These tests allowed us to determine the relationship between processing conditions and the material’s behavior during mold filling, as well as the quality of the resulting products.

## 3. Results and Discussion

### 3.1. Assessment of Mechanical Properties

Samples for testing mechanical properties and measuring water absorption were prepared using injection molding technology in accordance with the technological parameters recommended by the material manufacturer. During initial tests, necessary corrections were made to obtain high-quality, defect-free products. Table 2 presents the injection process parameters. Before processing, the EM20010 pellets were dried for 80 h at 60 °C. The extended drying time was due to the material being stored for an extended period before the final series of tests, which could have led to gradual absorption of moisture from the surrounding environment. The goal of this procedure was to ensure the complete removal of moisture and to guarantee identical initial conditions for all samples used in the experiment. The drying temperature was set at 60 °C, which is significantly below the processing temperature of the material and the range in which thermal degradation processes of PHA occur. Using a relatively low temperature with an extended drying time enabled effective moisture removal without exposing the material to thermal degradation. This is particularly important for biopolymers, as even low water content can initiate hydrolytic shortening of polymer chains during the injection molding process, leading to reduced mechanical properties and increased variability in test results.

According to literature data, thermal degradation of polyhydroxyalkanoates occurs primarily at processing temperatures, typically exceeding 170–180 °C. Therefore, the adopted temperature of 60 °C does not cause significant changes in the chemical structure of the material, while extended drying time ensures effective removal of moisture absorbed during storage and increases the repeatability of the conducted tests [56,57,61].

Before starting the tests to determine the mechanical properties, all samples in the form of beams and dumbbells were conditioned at a constant temperature of 23 °C and humidity of 50% for 48 h.

**Figure 3:**
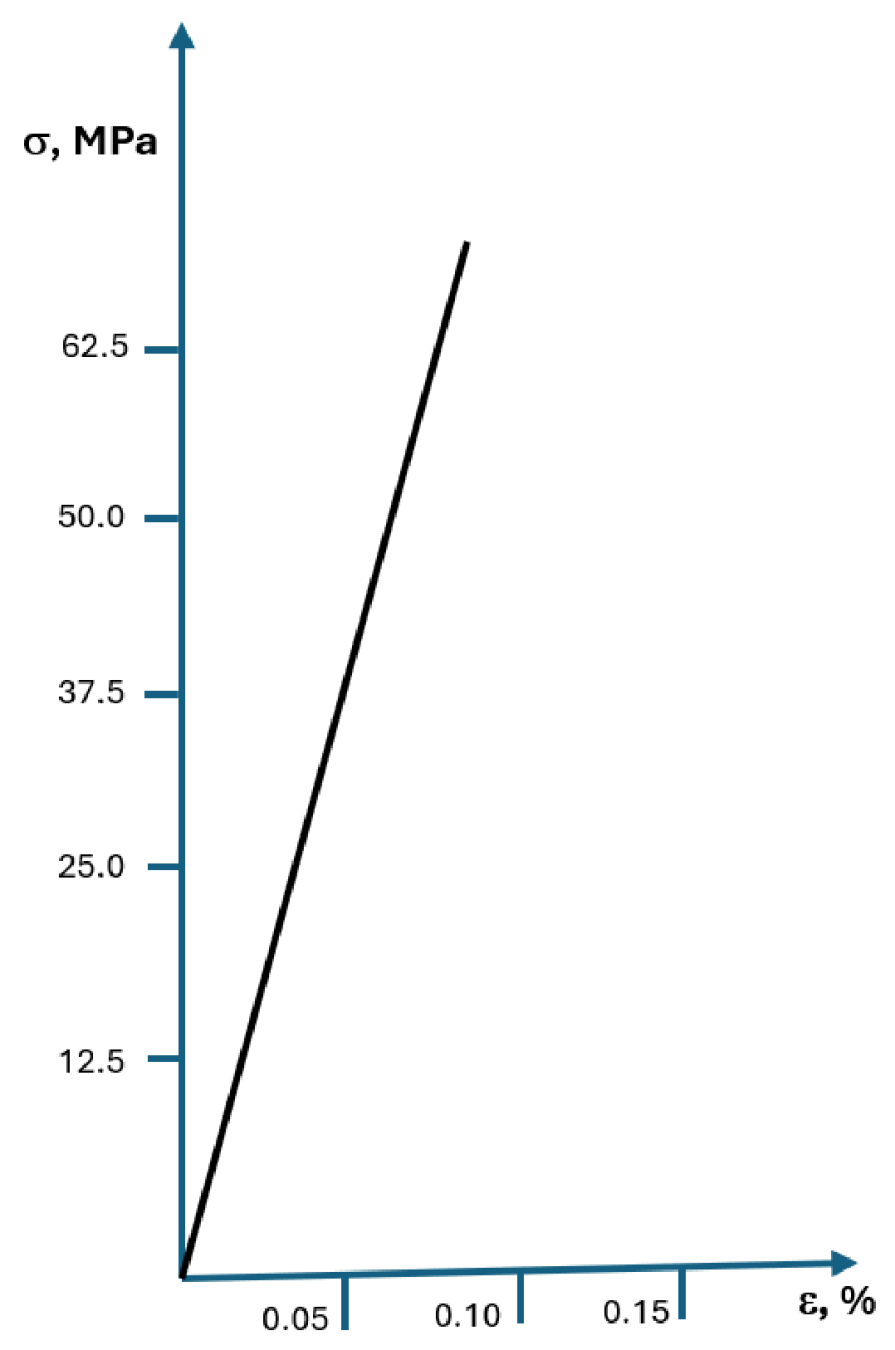
Example of the maximum tension curve of the tested material.

The test results obtained during the static tensile test are summarized in Table 3, while Figure 3 shows an example stress–strain curve obtained for the tested material. The tests were conducted in accordance with the requirements of ISO 527-2 [62].

Five measurement repetitions were performed for each variant, allowing for statistical analysis of the results obtained. The number of samples was selected to ensure that the obtained mean values were characterized by an acceptable level of dispersion. Based on the obtained data, mean values of individual mechanical parameters and their corresponding standard deviations were determined.

To determine the reliability of the results, a measurement uncertainty analysis was conducted. Expanded uncertainty was determined based on the standard deviation using the student’s t-distribution, assuming a 95% confidence level. This approach allowed for the estimation of the range within which the actual values of the tested mechanical parameters lie with a high degree of probability and enabled the assessment of the repeatability of the conducted tests.

Repeated measurements demonstrated high repeatability of the results. All analyzed samples yielded similar stress–strain curves, characterized by a nearly linear increase in stress with increasing strain until sample failure (Figure 3). This pattern indicates a dominant contribution of elastic deformation and a limited capacity of the material for plastic deformation.

The static tensile test results indicate that the EM20010 material exhibits a favorable combination of relatively high stiffness and mechanical strength with limited deformation capacity. The Young’s modulus of 1322.00 ± 20.62 MPa indicates significant stiffness. This is like values observed for some commercial PHA blends with thermoplastic starch (TPS) modified with biodegradable polyesters, and lower than typical values obtained for polylactide (PLA), for which the elastic modulus typically ranges from approximately 2500 to 3500 MPa, and for some PHB and PHBV grades, for which it can reach values of 2000 to 4000 MPa. The tensile strength of 66.80 ± 3.81 MPa obtained in this study is relatively high compared to many biodegradable polymers. It exceeds typical values obtained for thermoplastic starch (TPS), which are most often in the range of about 5–20 MPa, and is within the range of values characteristic for high-quality PLA, for which the tensile strength can be about 50–70 MPa. The obtained value is also higher than values often reported for commercial PHBV grades (about 20–40 MPa) and is in the upper range of values reported for PHB, whose tensile strength can reach 30–60 MPa, and in the case of materials with a sufficiently developed crystalline structure, it can exceed 60 MPa.

It should also be emphasized that the experimentally obtained tensile strength (66.80 MPa) is higher than the 35–45 MPa range declared by the manufacturer for the EM20010 material. This discrepancy should be interpreted with caution, as the mechanical properties of polymer materials may depend on factors such as processing conditions, sample preparation method, conditioning conditions, and parameters of the test method used. One possible explanation for the observed difference may be the different processing and sample preparation conditions used in this study compared to those used by the manufacturer when determining the material properties. However, based on the conducted tests, it is impossible to unequivocally conclude that these factors are the direct cause of the discrepancy. Differences between material batches and the specific conditions under which the determination was performed may also influence the obtained values.

The strain at maximum stress of 5.00 ± 0.45% indicates the limited capacity of the tested material to undergo plastic deformation. This behavior is characteristic of many semi-crystalline biopolyesters, such as PLA, PHB, and PHBV, which combine relatively high stiffness and strength with limited deformation before failure. This material character is also confirmed by the nearly linear stress–strain curves obtained during the tests.

The low standard deviations obtained for the analyzed mechanical parameters indicate high repeatability of the test results and good repeatability of the sample preparation process. The limited scatter in the results suggests that the sample preparation conditions used ensured comparable material condition across the samples. However, the mechanical results alone do not provide a clear assessment of the material’s microstructural homogeneity, so this conclusion should be based primarily on the repeatability of the obtained mechanical properties.

In summary, the obtained results indicate that the tested EM20010 material combines relatively high strength and stiffness with limited but sufficient deformation capacity, which may make it an interesting material for packaging and technical applications requiring adequate stiffness and mechanical strength. At the same time, its application potential should be considered considering the specificity of the tested material type, the processing conditions used, and the fact that the presented results concern samples produced under a single, reference processing state.

Impact strength tests were conducted using the Charpy impact method. Measurements were taken using a pendulum impactor equipped with a 4J impact energy hammer. Measurements were performed on standardized, unnotched samples, which allowed for the assessment of the material’s ability to absorb energy during dynamic loading. The results are summarized in Table 4.

Charpy impact strength test results showed that the PHA material tested exhibited moderate energy absorption during dynamic loading. The average impact strength was 24.8 ± 3.5 kJ/m^2^, indicating relatively good resistance to sudden impact loads, particularly considering its biodegradable nature and the high stiffness previously confirmed in static tensile tests. Individual measurement values ranged from 18.8 to 31.3 kJ/m^2^. The observed scatter in the results is typical of impact strength tests of polymer materials, whose fracture mechanism is strongly influenced by local inhomogeneities in the material structure, macromolecular orientation, and the presence of micro-defects created during processing. Despite this, the standard deviation of 1.5 kJ/m^2^ and the relatively small measurement error demonstrate good repeatability of the tests and the homogeneity of the resulting molded parts.

Comparing the obtained results with the mechanical characteristics of the tested material, PHA exhibits a typical compromise between stiffness, strength, and resistance to dynamic loads. The absence of notches in the tested samples allowed for the assessment of the material’s actual fracture toughness without additional stress concentration. It can be assumed that the presence of the PLA additive and appropriately selected processing conditions contributed to the material’s favorable structure, ensuring relatively high impact strength.

The next measurement to assess mechanical properties was hardness. The Shore method and the D scale, adapted for hard thermoplastics, were used. Hardness measurement is one of the basic criteria characterizing resistance to local surface deformation. Twenty measurements were performed for each sample. The obtained results were statistically analyzed to determine the mean value and assess the dispersion of the measurement results. Based on the collected data, the standard deviation was determined and used to estimate the measurement uncertainty. The analysis was performed at an assumed confidence level of 95%, using the student’s t-distribution appropriate for the number of measurements performed. The expanded uncertainty value was calculated using a coverage factor of k = 2, which corresponds to a coverage probability of approximately 95%. The statistical analysis allowed us to determine the reliability of the obtained results and to estimate the range within which the actual hardness value of the tested material lies with a high degree of probability. The measurement results for the three samples are presented in Table 5.

The mean hardness values for individual samples were 55.15 Shore D, 55.40 Shore D, and 54.60 Shore D, respectively, indicating minor differences between the analyzed moldings. The observed differences between the mean values did not exceed 1 Shore D unit, Low standard deviations, ranging from 0.1888 to 0.2102 Shore D, confirm the very small scatter of results obtained within each sample. At the same time, the expanded uncertainty determined for the 95% confidence level (k = 2) did not exceed ±0.44 Sh “D”. The average hardness of the tested material, approximately 55 Sh “D,” indicates moderately high resistance to local surface deformation. Combined with the previously obtained static tensile and impact strength test results, this confirms the favorable balance of mechanical properties of the tested polyhydroxyalkanoate.

Of note is the fact that all samples were produced using the injection molding method. The very small variation in hardness results between individual molded parts demonstrates the high repeatability of the technological process and the homogeneous structure of the material obtained during processing. The obtained results confirm the correct selection of injection parameters, such as plasticization temperature, mold temperature, pressure, and injection speed. The lack of significant differences in hardness values also indicates that no phenomena occurred during processing that could lead to material degradation or the formation of structural inhomogeneities that could negatively impact the functional properties of the molded parts. The obtained results confirm that the applied technological conditions enabled the production of products with stable and repeatable mechanical properties, which is crucial for the industrial use of the tested material.

### 3.2. Water Absorption Tests

The water absorption of the tested material was assessed in accordance with the requirements of ISO 62. This parameter, also referred to as water absorption, measures the material’s ability to absorb water and is expressed as the percentage increase in the sample’s mass relative to its initial dry mass after a specified period of exposure to an aqueous environment. Water absorption testing is particularly important for biodegradable polymers, as the presence of moisture can affect both their mechanical properties and the degradation processes occurring during use.

The tests were performed using dumbbell samples made by injection molding. Before measurements began, the samples were conditioned under controlled temperature and humidity conditions for 24 h, and their initial mass was then determined. After initial measurements, the samples were placed in a container filled with water and stored in a climatic chamber maintained at 23 ± 2 °C. During the exposure, the samples were properly arranged so that they did not touch each other or the vessel walls, allowing free water access to the entire material surface. The immersion time was 24 h, in accordance with the recommendations of the ISO 62 standard. After the exposure, the samples were removed from the water, surface-dried with absorbent paper to remove excess water not bound to the material, and their mass was determined again. Based on the difference between the initial and final masses, the water absorption value of the tested polyhydroxyalkanoate was determined. The results of this measurement are summarized in Table 6. The water absorption was then determined using Formula (1).

*X* = ((*m*_2_ − *m*_1_)/*m*_1_) × 100% (1)

where

*X*—water absorption (water absorption), %;

*m*_1_—dry sample mass, g;

*m*_2_—sample mass after soaking in water, g.

The final water absorption results are summarized in Table 7.

**Table 6:** Weight measurement result for samples before and after soaking.

Measurement No.	Sample No. 1		Sample No. 2		Sample No. 3	
	Mass Before Soaking	Weight After 24 h of Soaking	Mass Before Soaking	Weight After 24 h of Soaking	Mass Before Soaking	Weight After 24 h of Soaking
1	10.479	10.490	10.485	10.500	10.501	10.512
2	10.479	10.491	10.486	10.499	10.503	10.515
3	10.478	10.490	10.487	10.497	10.504	10.515
4	10.478	10.490	10.486	10.498	10.503	10.515
5	10.477	10.491	10.484	10.499	10.505	10.505
Final result	10.4782 ± 0.0010	10.4904 ± 0.0007	10.4782 ± 0.0014	10.4986 ± 0.0014	10.5032 ± 0.0018	10.5124 ± 0.0054

**Table 7:** Water absorption results of samples.

Sample No.	Difference in Mass, g	Water Absorption, %
Sample No. 1	0.0122	0.12
Sample No. 2	0.0132	0.12
Sample No. 3	0.0092	0.09
Result (average value)	0.0115	0.11

The water absorption test results presented in Table 6 and Table 7 indicate that the tested EM20010 material exhibits low water absorption capacity over short exposure periods. After 24 h of water immersion, the average weight gain of the samples was 0.0115 g, corresponding to an average water absorption of 0.11%. Analysis of individual samples demonstrated high repeatability of the obtained results—water absorption values ranged from 0.09 to 0.12%, with small differences between samples. This demonstrates the homogeneity of the tested material and the good repeatability of the injection molding process used to produce the samples. The small scatter in the results may also indicate the absence of significant structural discontinuities, such as micropores or air voids, that could promote increased moisture absorption.

The obtained water absorption value is relatively low compared to many other biodegradable materials, indicating limited water absorption during the initial period of contact with a humid environment. From a performance perspective, this is a beneficial characteristic, as low moisture absorption can promote dimensional stability of components and reduce the risk of changes in mechanical properties during short-term use in conditions of elevated humidity.

Water absorption tests are particularly important for polyhydroxyalkanoates, as the presence of water is one of the factors initiating hydrolytic processes in biodegradable polymers. Assessing a material’s ability to absorb water allows us to determine its behavior at an early stage of contact with moisture and is an important element of material characterization.

It should be emphasized, however, that the presented results only concern 24-h water absorption and reflect the material’s short-term resistance to exposure to an aqueous environment. They do not provide any conclusions about long-term mechanical stability, degradation processes, or the material’s behavior after the end of its service life. Assessing these properties requires long-term aging and degradation tests, including extended exposure times and analysis of changes in the material’s mechanical and structural properties. Therefore, the obtained results should be interpreted solely as a characteristic of the short-term water absorption of the tested material.

### 3.3. Molding Flow Test

**Figure 4:**
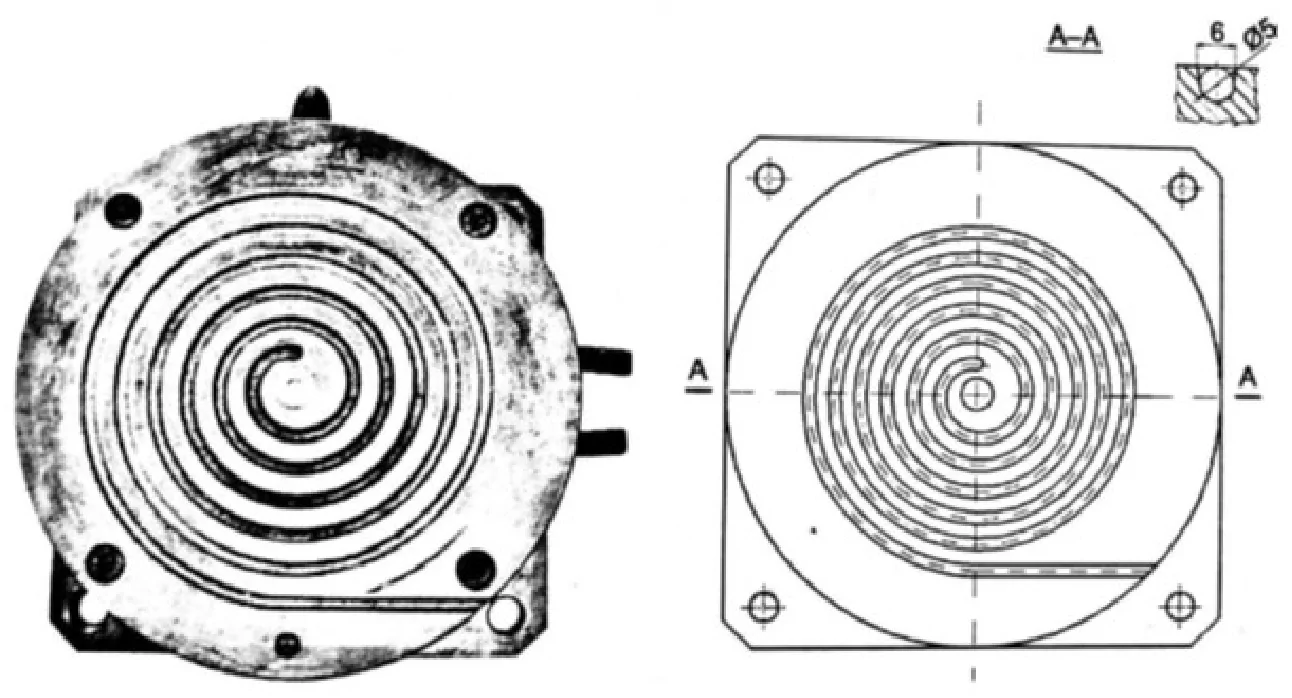
Schematic diagram of an injection mold with a spiral cavity arrangement [63].

To test the fluidity of the tested material, a technological flow test was conducted using an Archimedean spiral-shaped mold with an open mold cavity. This means that the cavity space is not completely enclosed and air can freely escape during the injection process, eliminating the need for additional venting systems. A diagram of the mold cavity is shown in Figure 4. The material supply system consisted of a 40 mm long inlet channel with a circular cross-section of 5 mm diameter (u-shaped), ensuring a uniform flow of the plasticized material into the mold cavity. The main sprue was designed as a 3° cone, facilitating its separation from the stationary part of the mold after the injection process and ensuring correct removal of the molded part. The geometry of the sprue system ensured stable material flow conditions during the flow test [63].

The tests were conducted under specified process parameters, including melt temperature, maximum injection pressure, and a specified pressure exposure time. After this time, the injection pressure was reduced to zero, thus ceasing further material flow in the mold cavity.

The primary parameter measured during the test was the length of the resulting spiral molded part, which provides a direct measure of the material’s ability to flow under the conditions of the actual injection molding process. Additionally, the mass of the resulting molded parts was also measured, which served as an auxiliary parameter in assessing the processing properties of the tested material.

This method is similar in its assumptions to the determination of the melt flow rate (MFR), but unlike standard rheological tests, it allows for the assessment of material behavior under conditions corresponding to the actual processing process. Although the spiral flow test is not covered by standard requirements, it is commonly used as a technological test to compare the processing properties of different materials and to assess the effect of process parameters on the ability to fill the mold cavity [61,63].

**Figure 5:**
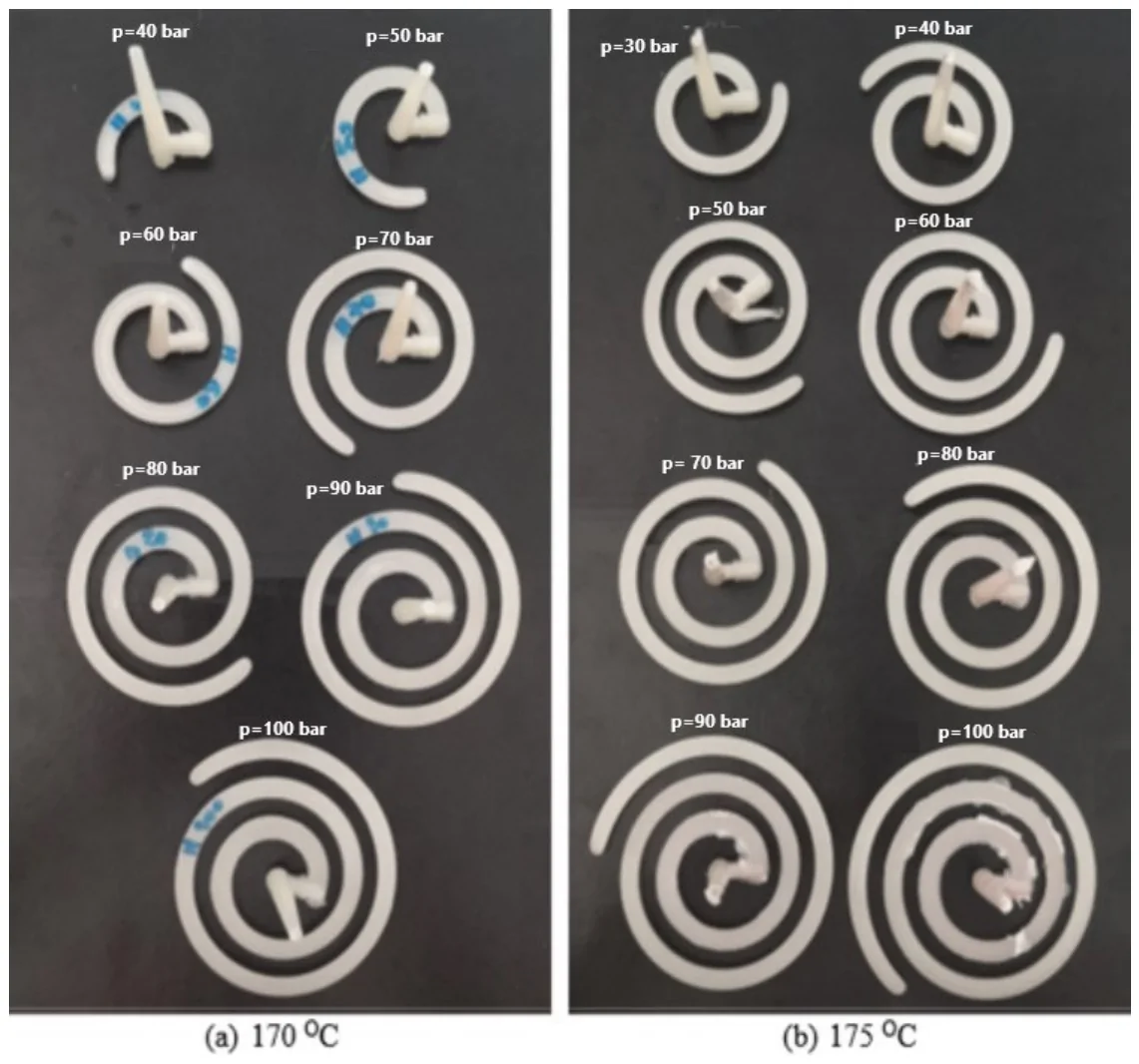
View of individual moldings depending on injection temperature and pressure.

Assuming the technological parameters shown in Table 2, two process temperatures were selected for fluidity measurements: 170 °C and 175 °C. The following injection pressures were assumed: 30, 40, 50, 60, 70, 80, 90, and 100 bar. Images of individual samples are shown in Figure 5, while the spiral mass and length measurements are presented in Table 8.

Analysis of the results of the technological flow test revealed a significant effect of both processing temperature and injection pressure on the flowability of the tested polyhydroxyalkanoate. As both parameters increased, a systematic increase in the length and weight of the resulting spiral moldings was observed, demonstrating an improvement in the material’s ability to fill the mold cavity (Figure 5).

At an injection temperature of 170 °C and a pressure of 30 bar, no molded part was obtained, indicating that the energy supplied to the system was insufficient to overcome the flow resistance of the molten polymer. Only by increasing the pressure to 40 bar did the spiral channel partially fill, resulting in a molded part 6.5 cm long. At subsequent measurement points, a significant increase in spiral length was observed, reaching a maximum of 47.2 cm at a pressure of 100 bar.

A similar trend was observed at 175 °C, but in this case, spiral lengths were greater across the entire pressure range tested. At a pressure of 30 bar, a 13.8 cm long compact was obtained, while at the maximum pressure of 100 bar, the spiral length reached 54.8 cm. This means that increasing the processing temperature by just 5 °C significantly reduced the melt’s viscosity and improved its flowability.

**Figure 6:**
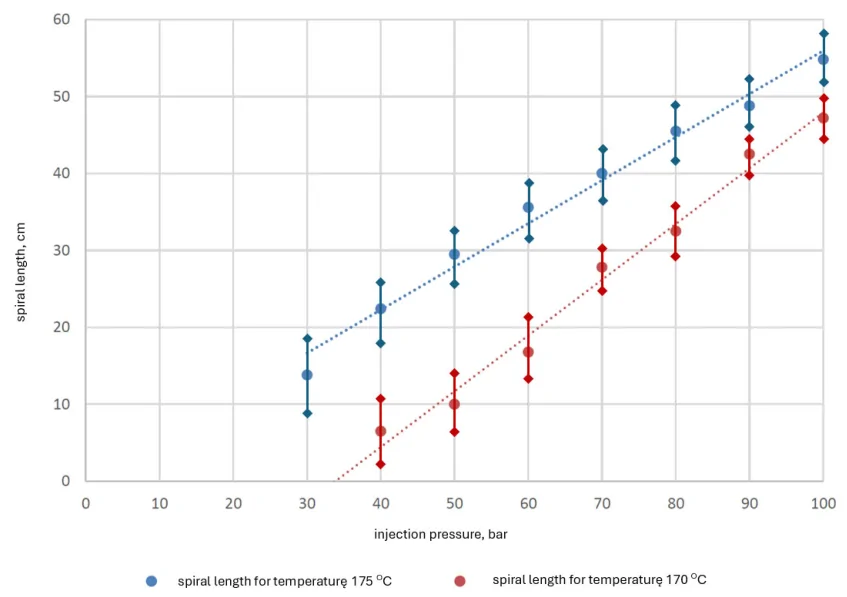
Length of spiral molding depends on injection pressure for different temperatures.

**Figure 7:**
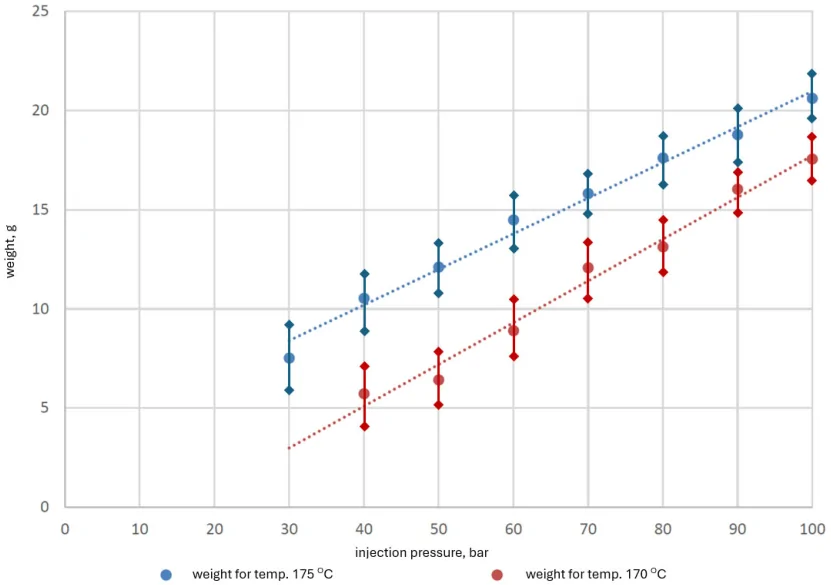
Mass of spiral moldings depending on injection pressure for different temperatures.

Comparison of the results obtained for both temperatures indicates that the effect of temperature was particularly pronounced at lower injection pressures. For example, at 40 bar, the spiral length increased from 6.5 cm at 170 °C to 22.4 cm at 175 °C, representing a more than threefold increase in flowability. As pressure increased, the differences between temperatures gradually decreased, but even at 100 bar, the spiral length at 175 °C was approximately 16% greater than at 170 °C. A comparison of the results for the change in spiral length and mass is presented in Figure 6 and Figure 7.

Similar relationships were observed during the analysis of the molded part mass (Figure 7). For both temperatures, increasing injection pressure led to a systematic increase in the mass of the resulting products, indicating increasingly complete mold filling. The maximum molded part mass was obtained at 175 °C and 100 bar, at 20.62 g. For the same pressure conditions but lower processing temperature, the molded part mass was lower, at 17.56 g.

The results obtained clearly confirm that the tested material is significantly sensitive to processing conditions. Both increasing the melt temperature and increasing the injection pressure lead to improved material fluidity, enabling more effective filling of the mold cavity. This phenomenon is typical of thermoplastics, whose viscosity decreases with increasing temperature and shear rate during flow through the gating system.

From a technological perspective, the obtained results indicate that the most favorable processing conditions for the tested polyhydroxyalkanoate occur at an injection temperature of 175 °C and pressures in the range of 90–100 bar. These parameters ensure the highest material fluidity (maximum spiral length), leading to the most complete mold filling. This is crucial when manufacturing products with complex geometries and thin-walled components. These results also confirm the correct selection of the range of technological parameters used during the research and indicate the material’s good processing properties via injection molding.

## 4. Conclusions

The conducted research allowed for the evaluation of selected processing and performance properties of commercial polyhydroxyalkanoate-based material intended for injection molding. The obtained results confirmed that the material exhibits favorable mechanical properties and low short-term water absorption after processing under the manufacturer’s recommended conditions. This demonstrates its suitability for manufacturing products requiring adequate strength and stability under typical operating conditions. However, it should be emphasized that the presented results refer to the material processed using a single, reference set of technological parameters and do not constitute an assessment of the impact of injection molding conditions on its mechanical properties.

A significant element of the work was the analysis of processing properties using a technological flow test conducted in a spiral mold. It was demonstrated that processing temperature and injection pressure significantly affect the material’s ability to flow and fill the mold cavity. The results obtained confirm that the proper selection of process parameters plays a key role in ensuring the correct operation of the injection molding process and obtaining molded parts of appropriate quality.

The conducted research provided characteristics of selected functional and processing properties of the tested material, providing a valuable addition to knowledge regarding the processing capabilities of commercial PHA-based biopolymers. The obtained results can serve as a basis for further research, including assessing the impact of injection molding process parameters on the mechanical properties and structure of the material, as well as analyzing its long-term durability and behavior under operating and degradation conditions.

## Figures and Tables

**Table 1:** Properties of the tested polyhydroxyalkanoate EM20010 [49].

Parameter	Researcher’s Method	Value/Unit
Density	ISO 1183	1.33 g/cm^3^
Melt flow rate, MFR (2.16 kg, 190 °C)	ISO 1133	8 g/10 min
Softening point (Vicat A/120)	ISO 306	105 °C
Melting point	ISO 11357	150–175 °C
Processing shrinkage	-	0.3–1.2%
Water absorption	ISO 62	<0.4%
Maximum tension	ISO 527	35–45 MPa
Young’s modulus	ISO 527	1650–2000 MPa
Elongation at break	ISO 527	3–8%
Flexural modulus	ISO 178	2500–3500 MPa
Bending strength	ISO 178	45–75 MPa
Izod impact strength (notched)	ISO 180	2.5–4.5 kJ/m^2^

**Table 2:** Parameters of the injection process of test samples.

Injection Parameters		Values
injection:		
speed		40 mm/s
pressure		150 bar
processing temperature	zone 1	180 °C
	zone 2	175 °C
	zone 3	160 °C
	zone 4	145 °C
holding pressure:		
time		8 s
clamping pressure		80 bar
closing force		
mean		844 N
closing the mold		
pressure		170 bar
speed		30 mm/s
mold protection time		10 s
cycle time		120 s
counter pressure		5 bar
opening the mold		
counter pressure		150 bar
cooling time		20 s
temperature		25 °C

**Table 3:** Summary of results from the static tensile test.

Relative Deformation at Maximum Stress ε, %	Maximum Tension σ, MPa	Young’s Modulus E, MPa
5.00 ± 0.45	66.80 ± 3.81	1322.00 ± 20.62

**Table 4:** Impact strength measurement result.

Measurement No.	The Energy of Destruction, J	Measurement Result, Impact Strength, kJ/m^2^
1	1.00	25.0
2	1.25	31.3
3	0.85	21.3
4	0.85	21.3
5	0.75	18.8
6	1.25	31.3
7	1.25	31.3
8	0.85	21.3
9	1.00	25.0
10	0.85	21.3
Average value		24.8
Standard deviation (k = 2, N = 95%)		1.5
Measurement error		±3.5
Result		24.8 ± 3.5

**Table 5:** Hardness measurement result after processing.

Measurement No.	Sample No. 1, Sh “D”	Sample No. 2, Sh “D”	Sample No. 3, Sh “D”
1	54.5	57.0	55.5
2	56.0	56.5	54.0
3	56.5	55.5	53.5
4	55.5	54.5	54.5
5	55.5	54.5	53.5
6	56.0	55.0	54.0
7	55.5	54.5	55.5
8	54.0	56.0	55.5
9	54.5	56.5	55.5
10	55.0	54.5	54.0
11	55.5	56.0	55.5
12	55.0	55.5	53.0
13	56.5	55.0	54.5
14	55.0	57.0	54.0
15	54.5	54.5	55.5
16	53.5	54.0	53.5
17	55.5	55.0	54.5
18	54.0	54.5	55.5
19	54.5	55.5	54.5
20	56.0	56.5	56.0
Average value	55.15	55.40	54.60
Standard deviation	0.1888	0.2102	0.2006
Measurement error (k = 2, NP = 95%)	±0.40	±0.44	±0.42
Result	55.15 ± 0.40	55.40 ± 0.44	54.60 ± 0.42

**Table 8:** Spiral mass and length depending on injection pressure and temperature.

Injection Pressure, bar	Injection Temperature: 170 °C		Injection Temperature: 175 °C	
	Mass, g	Spiral Length, cm	Mass, g	Spiral Length, cm
30	-	-	7.52	13.8
40	5.73	6.5	10.53	22.4
50	6.42	10.0	12.11	29.5
60	8.90	16.8	14.49	35.6
70	12.08	27.8	15.82	40.0
80	13.13	32.5	17.62	45.5
90	16.04	42.5	18.79	48.8
100	17.56	47.2	20.62	54.8

## Data Availability

The original contributions presented in this study are included in the article. Further inquiries may be directed to the corresponding author.

## References

[1] Marano S., Laudadio E., Minnelli C., Stipa P. (2022). Tailoring the Barrier Properties of PLA: A State-of-the-Art Review for Food Packaging Applications. Polymers.

[2] Kabir E., Kaur R., Lee J., Kim K.H., Kwon E.E. (2020). Prospects of biopolymer technology as an alternative option for non-degradable plastics and sustainable management of plastic wastes. J. Clean. Prod..

[3] Gigante V., Cinelli P., Seggiani M., Alavarez V.A., Lazzeri A., Koller M. (2020). Processing and thermomechanical properties of PHA. The Handbook of Polyhydroxyalkanoates.

[4] Lalonde J.N., Pilania G., Marrone B.L. (2025). Materials designed to degrade: Structure, properties, processing, and performance relationships in polyhydroxyalkanoate biopolymers. Polym. Chem..

[5] Attaran S.A., Hassan A., Wahit M.U. (2015). Materials for food packaging applications based on bio-based polymer nanocomposites: A review. J. Thermoplast. Compos. Mater..

[6] Sotiropoulos N.P., Mindrinos L., Peltier J.-D., Filippou K.V., Kotzabasaki M.I., Tsigkas N., Maraveas C. (2026). Machine Learning Prediction of Thermal Properties of PHB/PHBV-Based Materials: A Quantitative Structure–Property Relationship Approach Using an Integrated Polymer Database. Polymers.

[7] Das R., Saha N.R., Pal A., Chattopadhyay D., Paul A.K. (2018). Comparative Evaluation of Physico-Chemical Characteristics of Biopolyesters P(3HB) and P(3HB-Co-3HV) Produced by Endophytic Bacillus Cereus RCL 02. Front. Biol..

[8] Fabijański M., Garbarski J. (2026). The Influence of Cellulose Fiber Content on the Mechanical Properties of Composites Based on Modified Thermoplastic Starch. Processes.

[9] Mosca Balma A., Meinardi S., Roato I., Mussano F. (2026). Polyhydroxyalkanoates in Bone Alloplastic Materials: State of the Art and Future Perspectives. Polymers.

[10] Zhang Z., Li J., Ma L., Yang X., Fei B., Leung P.H.M., Tao X. (2020). Mechanistic Study of Synergistic Antimicrobial Effects between Poly (3-Hydroxybutyrate) Oligomer and Polyethylene Glycol. Polymers.

[11] Verble J.A., McKinney M.L. (2026). Microbial Degradation of Plastics in Freshwater Environments. Microplastics.

[12] Kim M.-J., Kim H., Park M.C., Kim S.-B., Jang D.-J., Kim S.T. (2026). Polyhydroxyalkanoate-Based Microparticles for Enhanced Photostability and Controlled Release of Pyraclostrobin. Polymers.

[13] Garbarski J., Fabijański M. (2025). Application of a Filler in the Form of Micronized Chalcedonite to Biodegradable Materials Based on Thermoplastic Starch as an Element of the Sustainable Development of Polymeric Materials. Sustainability.

[14] Raut S.S., Sharma A., Mishra A. (2026). A decade of bibliometric and biotechnological advances in microbial polyhydroxyalkanoate (PHA) for biomedical applications: Developments in economical production, biosynthetic pathways, physicochemical properties, and therapeutic potential: A review. Int. J. Biol. Macromol..

[15] Costa P., Conti A., Paulon V., Corte L., Cardinali G., Casella S., Kennes C., Veiga M.C., Basaglia M., Favaro L. (2026). Monitoring Hydrogen-Producing Bacterial Consortia During Acidogenesis of Fruit Waste Towards Autotrophic and Heterotrophic Polyhydroxyalkanoate Production. Appl. Sci..

[16] https://www.plastix.it/al-via-la-partnership-tra-gammarad-italia-e-bio-on/.

[17] Thamarai P., Vickram A.S., Saravanan A., Deivayanai V.C., Evangeline S. (2024). Recent Advancements in Biosynthesis, Industrial Production, and Environmental Applications of Polyhydroxyalkanoates (PHAs): A Review. Bioresour. Technol. Rep..

[18] Brojanigo S., Alvarado-Morales M., Basaglia M., Casella S., Favaro L., Angelidaki I. (2022). Innovative Co-Production of Polyhydroxyalkanoates and Methane from Broken Rice. Sci. Total Environ..

[19] Saratale R.G., Cho S., Saratale G.D., Kumar M., Bharagava R.N., Varjani S., Kadam A.A., Ghodake G.S., Palem R.R., Mulla S.I. (2021). An Overview of Recent Advancements in Microbial Polyhydroxyalkanoates (PHA) Production from Dark Fermentation Acidogenic Effluents: A Path to An Integrated Bio-Refinery. Polymers.

[20] Fabijański M. (2026). Assessment of global migration and sensory properties of modified polylactide packaging. Przem. Chem..

[21] Garcia-Gonzalez L., De Wever H. (2018). AceticAcid as an Indirect Sink of CO2 for the Synthesis of Polyhydroxyalkanoates (PHA): Comparison with PHA Production Processes Directly Using CO_2_ as Feedstock. Appl. Sci..

[22] Colombo B., Sciarria T.P., Reis M., Scaglia B., Adani F. (2016). Polyhydroxyalkanoates (PHAs) production from fermented cheese whey by using a mixed microbial culture. Bioresour. Technol..

[23] Zhang D., Jiang H., Chang J., Sun J., Tu W., Wang H. (2019). Effect of thermal hydrolysis pretreatment on volatile fatty acids production in sludge acidification and subsequent polyhydroxyalkanoates production. Bioresour. Technol..

[24] Ye J., Huang W., Wang D., Chen F., Yin J., Li T., Zhang H., Chen G.-Q. (2018). PilotScale-up of Poly(3-hydroxybutyrate-co-4-hydroxybutyrate) Production by Halomonas bluephagenesis via Cell Growth Adapted Optimization Process. Biotechnol. J..

[25] Kosseva M.R., Rusbandi E. (2018). Trends in the biomanufacture of polyhydroxyalkanoates with focus on downstream processing. Int. J. Biol. Macromol..

[26] Raza Z.A., Abid S., Banat I.M. (2018). Polyhydroxyalkanoates: Characteristics, production, recent developments and applications. Int. Biodeterior. Biodegrad..

[27] https://en.wikipedia.org/wiki/Polyhydroxyalkanoates.

[28] Fabijański M., Garbarski J., Szymaniak Z. (2025). Modifying Polylactide with Powdered Cork Filler. Materials.

[29] Anjum A., Zuber M., Zia K.M., Noreen A., Anjum M.N., Tabasum S. (2016). Microbial production of polyhydroxyalkanoates (PHAs) and its copolymers: A review of recent advancements. Int. J. Biol. Macromol..

[30] Jusic J., Filieri A., Crognale S., Manni M., Tamantini S., Vinciguerra V., Cardarelli A., Barbanera M., Jones D., Matt D. (2026). Chestnut Wood Residues, with and Without Tannins, as a Potential Feedstock for PHA Bioplastic Production. Polymers.

[31] Sharma V., Sehgal R., Gupta R. (2021). Polyhydroxyalkanoate (PHA): Properties and Modifications. Polymer.

[32] Zytner P., Kumar D., Elsayed A., Mohanty A., Ramarao B.V., Misra M. (2023). A Review on Polyhydroxyalkanoate (PHA) Production through the Use of Lignocellulosic Biomass. RSC Sustain..

[33] Keenan T.M., Nakas J.P., Tanenbaum S.W. (2006). Polyhydroxyalkanoate Copolymers from Forest Biomass. J. Ind. Microbiol. Biotechnol..

[34] Acharjee S.A., Bharali P., Gogoi B., Sorhie V., Walling B. (2023). Alemtoshi PHA-Based Bioplastic: A Potential Alternative to Address Microplastic Pollution. Water Air Soil Pollut..

[35] Mohan G., Johnson R.L., Yu J. (2021). Conversion of Pine Sawdust into Polyhydroxyalkanoate Bioplastics. ACS Sustain. Chem. Eng..

[36] Zhang L., Jiang Z., Tsui T.-H., Loh K.-C., Dai Y., Tong Y.W. (2022). A Review on Enhancing Cupriavidus Necator Fermentation for Poly(3-Hydroxybutyrate) (PHB) Production From Low-Cost Carbon Sources. Front. Bioeng. Biotechnol..

[37] Spinosa M., Savarino D.M., Demichelis F., Fino D., Lombardi P., Russo N., Todella E., Tommasi T. (2026). From Orange Waste to Biocomposite: Environmental Assessment of Orange-Peel Reinforced PHA Material. Sustainability.

[38] Tao X., Liu S., Lv L., Sun L., Zhang G., Liang J., Zou W. (2025). Recent advances in polyhydroxyalkanoate production from volatile fatty acids derived from food waste fermentation. Front. Microbiol..

[39] Amaro T.M.M.M., Rosa D., Comi G., Iacumin L. (2019). Prospects for the Use of Whey for Polyhydroxyalkanoate (PHA) Production. Front. Microbiol..

[40] Kusuma H.S., Sabita A., Putri N.A., Azlina N., Ilyanasafa N., Darmokoesoemo H., Amenaghawon A.N., Kurniawan T.A. (2024). Waste to wealth: Polyhydroxyalkanoates (PHA) production from food waste for a sustainable packaging paradigm. Food Chem. Mol. Sci..

[41] Koller M. (2026). Advances in Polyhydroxyalkanoate (PHA) Production. Bioengineering.

[42] Liang X., Cha D.K., Xie Q. (2024). Properties, production, and modification of polyhydroxyalkanoates. Resour. Conserv. Recycl. Adv..

[43] Fabijański M., Gołofit T. (2024). Influence of Processing Parameters on Mechanical Properties and Degree of Crystallization of Polylactide. Materials.

[44] Możejko-Ciesielska J., Kiewisz R. (2016). Bacterial polyhydroxyalkanoates: Still fabulous?. Microbiol. Res..

[45] Mozejko-Ciesielska J., Szacherska K., Marciniak P. (2019). Pseudomonas Species as Producers of Eco-friendly Polyhydroxyalkanoates. J. Polym. Environ..

[46] Sepúlveda M.I., Seeger M., Vidal G. (2025). Carbon recovery from wastewater feedstocks: Synthesis of polyhydroxyalkanoates for target applications. Resources.

[47] Da Cruz Pradella J.G., Koller M. (2021). Economics and industrial aspects of PHA production. The Handbook of Polyhydroxyalkanoates.

[48] Data Sheet: Ecomann Bioresin EM20010. https://www.specialchem.com/plastics/product/ecomann-ecomann-bioresin-em20010.

[49] Ienczak J.L., Schmidell W., de Aragão G.M.F. (2013). High-cell-density culture strategies for polyhydroxyalkanoate production: A review. J. Ind. Microbiol. Biotechnol..

[50] Fabijański M. (2022). Effect of multiple processing on the strength properties of polylactide/polystyrene mixture. Przem. Chem..

[51] Tryznowski M., Żołek-Tryznowska Z. (2020). Surface Properties of Poly (Hydroxyurethane)s Based on Five-Membered Bis-Cyclic Carbonate of Diglycidyl Ether of Bisphenol A. Materials.

[52] Núñez-Pérez J., Cornejo-Lucero O.J., Espin-Valladares R.C., Barba P., Rodríguez Cabrera H.M., Pais-Chanfrau J.-M. (2026). Production and Characterisation of Polyhydroxyalkanoates from Cocoa Mucilage Using a Wild-Type Priestia aryabhattai Strain. Processes.

[53] Getino L., Martín J.L., Chamizo-Ampudia A. (2024). A Review of Polyhydroxyalkanoates: Characterization, Production, and Application from Waste. Microorganisms.

[54] Vandi L.-J., Chan C.M., Werker A., Richardson D., Laycock B., Pratt S. (2018). Wood-PHA Composites: Mapping Opportunities. Polymers.

[55] Singh Saharan B., Grewal A., Kumar P. (2014). Biotechnological production of polyhydroxyalkanoates: A review on trends and latest developments. Chin. J. Biol..

[56] Katagi V.N., Bhat S.G., Paduvari R., Kodavooru D., Somashekara D.M. (2023). Waste to value-added products: An innovative approach for sustainable production of microbial biopolymer (PHA)-emphasis on inexpensive carbon feedstock. Environ. Technol. Rev..

[57] Abbas M.I., Amelia T.S.M., Bhubalan K., Vigneswari S., Ramakrishna S., Amirul A.A. (2025). Bioprospecting waste for polyhydroxyalkanoates production: Embracing low carbon bioeconomy. Int. J. Environ. Sci. Technol..

[58] (2019). Plastics—Determination of Tensile Properties—Part 1: General principles.

[59] (2023). Plastics—Determination of Charpy Impact Properties—Part 1: Non-Instrumented Impact Test.

[60] (2003). Plastics and Ebonite—Determination of Indentation Hardness by Means of a Durometer (Shore Hardness).

[61] Lackner M., Costa P., Koller M., Zinn M. (2024). More than PHB–the PHAmily of copolymers: Justifications for broader use and summary of biodegradation facts on polyhydroxyalkanoates (PHA)—A review. Chem. Biochem. Eng. Q..

[62] (2025). Plastics—Determination of Tensile Properties—Part 2: Test Conditions for Moulding and Extrusion Plastics.

[63] Wilczyński K., Nastaj A. (2024). Wybrane Zagadnienia z Przetwórstwa Tworzyw Sztucznych. Laboratorium (Selected Issues in Plastics Processing. Laboratory).

